# Transcriptome sequencing and whole genome expression profiling of chrysanthemum under dehydration stress

**DOI:** 10.1186/1471-2164-14-662

**Published:** 2013-09-28

**Authors:** Yanjie Xu, Shan Gao, Yingjie Yang, Mingyun Huang, Lina Cheng, Qian Wei, Zhangjun Fei, Junping Gao, Bo Hong

**Affiliations:** 1 Department of Ornamental Horticulture, China Agricultural University, Beijing 100193, China; 2 Boyce Thompson Institute, Cornell University, Ithaca, NY 14853, USA; 3 USDA Robert W. Holley Center for Agriculture and Health, Tower Road, Ithaca, NY 14853, USA

**Keywords:** Chrysanthemum, Dehydration stress, Gene expression, Pathways, RNA-seq, Transcriptome

## Abstract

**Background:**

Chrysanthemum is one of the most important ornamental crops in the world and drought stress seriously limits its production and distribution. In order to generate a functional genomics resource and obtain a deeper understanding of the molecular mechanisms regarding chrysanthemum responses to dehydration stress, we performed large-scale transcriptome sequencing of chrysanthemum plants under dehydration stress using the Illumina sequencing technology.

**Results:**

Two cDNA libraries constructed from mRNAs of control and dehydration-treated seedlings were sequenced by Illumina technology. A total of more than 100 million reads were generated and de novo assembled into 98,180 unique transcripts which were further extensively annotated by comparing their sequencing to different protein databases. Biochemical pathways were predicted from these transcript sequences. Furthermore, we performed gene expression profiling analysis upon dehydration treatment in chrysanthemum and identified 8,558 dehydration-responsive unique transcripts, including 307 transcription factors and 229 protein kinases and many well-known stress responsive genes. Gene ontology (GO) term enrichment and biochemical pathway analyses showed that dehydration stress caused changes in hormone response, secondary and amino acid metabolism, and light and photoperiod response. These findings suggest that drought tolerance of chrysanthemum plants may be related to the regulation of hormone biosynthesis and signaling, reduction of oxidative damage, stabilization of cell proteins and structures, and maintenance of energy and carbon supply.

**Conclusions:**

Our transcriptome sequences can provide a valuable resource for chrysanthemum breeding and research and novel insights into chrysanthemum responses to dehydration stress and offer candidate genes or markers that can be used to guide future studies attempting to breed drought tolerant chrysanthemum cultivars.

## Background

Chrysanthemum (*Chrysanthemum morifolium*) is one of the most important ornamental crops in the world and the second most valuable cut flower, only after rose [1,2]. Currently drought stress is one of the major factors limiting chrysanthemum production. Therefore improving drought tolerance of chrysanthemum is the main focused area in its current breeding programs. Previous studies on the model plant Arabidopsis show that plant tolerance to drought stress is a multigenic trait and during the process of plant responses to drought stress, a large number of genes are induced, which further cause a series of physiological and biochemical alterations, such as changes in photosynthesis, primary biosynthetic pathways, the respiration pathway and the antioxidation pathway [3]. Plant drought-responsive genes can be classified into two groups: those that directly protect plants against environmental stresses and those that regulate the expression of downstream target genes in the stress response [4]. The first group is mainly comprised of enzymes in the biosynthesis of various osmoprotectants, late-embryogenesis-abundant (LEA) proteins, antifreeze proteins, chaperones, and detoxification enzymes. The second group mainly includes transcription factors, protein kinases, and enzymes involved in the phosphoinositide metabolism [5].

Compared with the functional proteins, the transcription factors always act at the upstream position of the signal transduction and gene regulatory network, controlling a broad range of downstream genes; which makes them efficient in tolerating abiotic stress. To date, several transcription factors belonging to different transcription factor families, such as MYB, bZIP, AP2/EREBP and WRKY, have been implicated in the regulation of stress responses [6,7]. The protein kinases, including calmodulin dependent protein kinases (CDPKs), mitogen-activated protein kinases (MAPKs), receptor protein kinases (RPKs), and ribosomal protein kinases, are involved in the signal cascade amplification in response to different stress factors [8].

Despite significant progress during the past decade in aiming to understand the pathways affected by drought stress, limited information is available regarding pathway dynamics in chrysanthemum under drought stress. We previously reported that over-expression of *AtDREB1A*, an Arabidopsis gene encoding a dehydration-responsive element binding (DREB) protein, in chrysanthemum conferred strong tolerance to drought stress [9]. We further identified a total of 74 chrysanthemum unique transcripts induced by AtDREB1A under drought stress using suppression subtractive hybridization [10]. Several other reports described roles of stress-inducible transcription factors in regulation of drought stress tolerance in chrysanthemum. Overexpression of *CgDREBa* in chrysanthemum enhanced drought tolerance by enhancing the proline content and the superoxide dismutase (SOD) activity [11]. Constitutive expression of *CdICE1* in chrysanthemum improved drought tolerance through regulating the expression of *CgDREB* genes, antioxidant enzyme activities and the proline content [12]. However, to date no information is found about genome-wide expression profiling of chrysanthemum under dehydration stress due to the limited genomics and functional genomics resources that are currently available in chrysanthemum.

Most chrysanthemum cultivars are polyploid (2n = 4× = 36 or 2n = 6× = 54) and highly heterozygous [13]. The genome of *chrysanthemum morifolium* is estimated to be approximately 9.4 Gb (http://data.kew.org/cvalues/). Due to its large and complex genome and complicated genetic background, very few genomic and genetics resources are currently available for chrysanthemum, which is regarded as one of the major factors limiting chrysanthemum breeding and biology research. Recent rapid advances in next-generation sequencing (NGS) technologies and associated bioinformatics tools have revolutionized plant transcriptomics researches. These efficient, reliable and cost-effective sequencing technologies have been widely used to characterize the transcriptomes of plants, particularly those of non-model organisms without a reference genome, for gene discovery, marker development and understanding gene regulatory networks of important biological processes [14]. In rice, enhanced α- linolenic acid metabolism in drought-tolerant landraces/genotypes under drought conditions is in compliment with its drought tolerance capacity [15]. Genes involved in stomatal closure inhibition, ascorbate–glutathione pathway and ubiquitin–proteasome system in *Populus euphratica* are thought to specially modulate the drought stress responses [16]. Enrichment of apoptosis and cell death gene categories among the positively selected genes in a study on *Eucalyptus camaldulensis* specie were enriched under water stress conditions [17].

Recently, the transcriptome of *Chrysanthemum nankingense*, a diploid closely related species of chrysanthemum, was sequenced, and EST-SSR markers were identified from these newly acquired sequences and used to survey the polymorphism among different chrysanthemum cultivars [18]. However, till now, it is still largely unknown about transcriotome changes in response to dehydration in chrysanthemum.

In this study, we performed large-scale transcriptome sequencing of chrysanthemum plants under dehydration stress using the Illumina sequencing technology for the purposes of 1) generating a functional genomics resource for chrysanthemum to facilitate its research and breeding; and 2) obtaining a deeper understanding of the molecular mechanisms regarding chrysanthemum responses to dehydration stress. We generated a total of more than 100 million reads from normal and dehydration-treated chrysanthemum plants. These reads were assembled de novo into approximately 100 K unique transcripts which were further extensively annotated. Regulatory proteins, biochemical pathways were predicted from these assembled transcripts. We then compared global expression profiles of chrysanthemum between normal and dehydration stress conditions and identified a number of dehydration-responsive genes. Further detailed analysis of these genes provided some novel insights into chrysanthemum responses to dehydration stress and offered candidate genes or markers that can be used to guide future efforts attempting to breed drought resistant chrysanthemum cultivars.

## Results and discussion

### Sequencing and de novo assembly of chrysanthemum transcriptome

**Figure 1:**
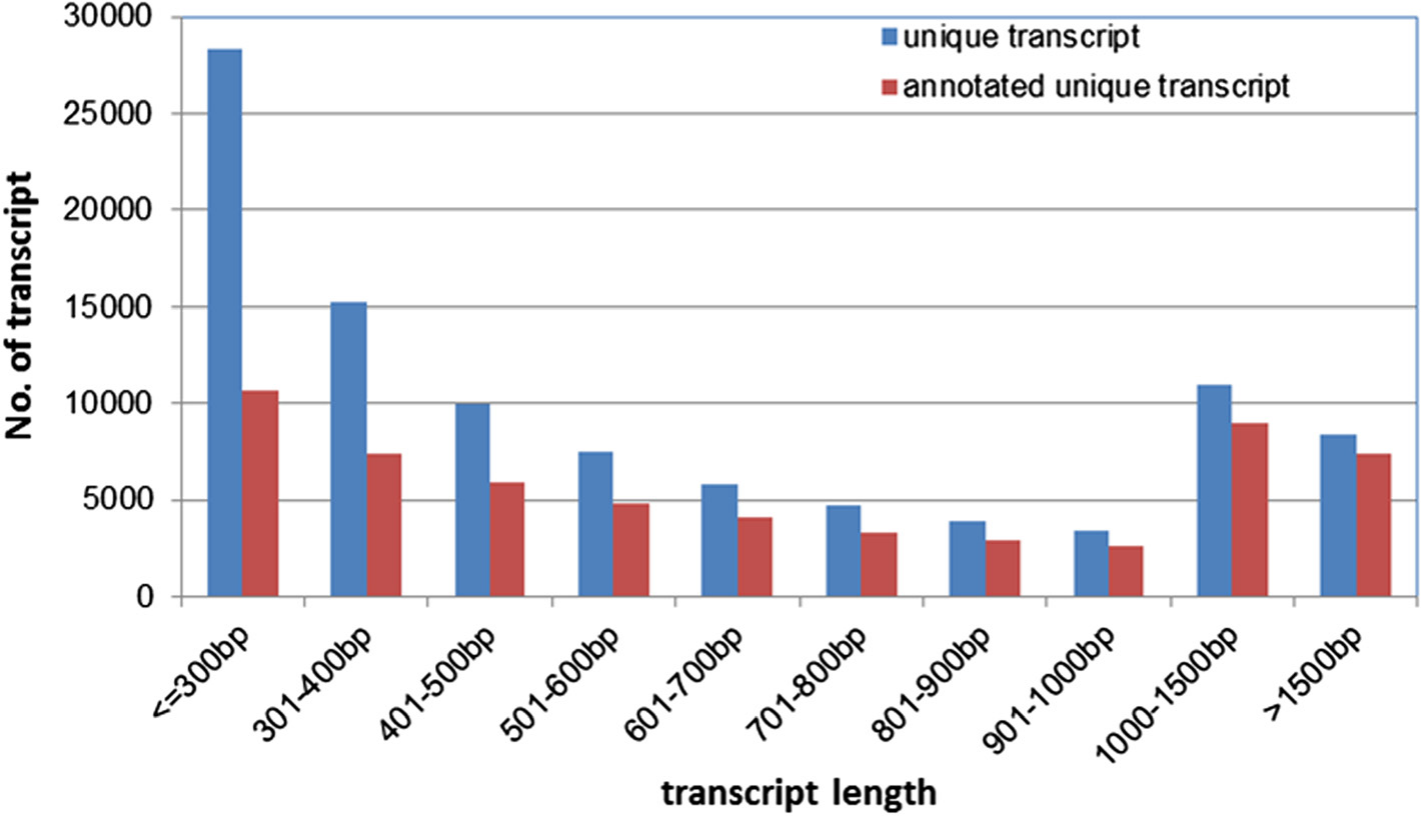
Length distribution of chrysanthemum unique transcripts.

Strand-specific RNA-seq libraries were prepared from whole plants of chrysanthemum [*Chrysanthemum morifolium* (Ramat) Kitamura] cv. Fall Color, under normal and dehydration conditions, respectively. Relative water content (RWC) of leaves was 94.4% in control plants, clearly contrasting with 53.9% RWC for samples collected at 3 h dehydration treatment. For each condition, three independent biological replicates were performed. And each RNA-seq library was sequenced twice on the Illumina HiSeq 2000 system, one with read length of 100 bp and one of 51 bp. After removing low quality, adaptor and barcode sequences, as well as possible virus and rRNA contaminated reads, a total of 52,254,807 and 55,748,055 reads of length 100 bp and 51 bp, respectively, were obtained (Table 1). De novo assembly of these high-quality cleaned reads generated 98,180 unique transcripts with an average length of 662.9 bp and the longest transcript of 8,877 bp. The length distribution of the assembled chrysanthemum unique transcripts is shown in Figure 1.

**Table 1:** Summary of chrysanthemum transcriptome sequencing dataset

**Items**	**Sequence**		** Total**
	**100 bp**	**51 bp**	
No. of reads	59,214,931	67,967,011	127,181,942
No. of cleaned reads	52,254,807	55,748,055	108,002,862
No. of mapped reads	38,925,881	47,706,654	86,632,535
No. of assembled transcripts			98,180
Average length of transcripts			662.9 bp
Total length of transcripts			65,085,887 bp

To efficiently distribute our transcriptome sequences and the associated analysis results to the research community and allow researchers to mine the chrysanthemum transcriptome dataset, we developed an online database called Chrysanthemum Transcriptome Database, which can be accessed at http://www.icugi.org/chrysanthemum. The database provides basic query and blast search functions and downloads to most of our analysis results.

Most chrysanthemum cultivars are highly heterozygous, including ‘Fall Color’ used in the present study. We scanned the chrysanthemum transcriptome and identified a total of 108,274 heterozygous sites (available at the Chrysanthemum Transcriptome Database), which represented approximately 0.17% of the assembled chrysanthemum transcriptome with a total size of 65,085,887 bp and 0.19% of the 56,288,302 bp used for this survey (covered by at least ten reads). Among these heterozygous sites, 67,668 (62.5%) contained two alleles involved in transition changes (AG or CT), 37,723 (34.8%) comprised two alleles involved in transversion changes (AC, AT, GC and GT), 2,872 (2.7%) contained two alleles involved in insertion/deletion (indel) changes and 10 had three different alleles. Among 98,180 assembled unique transcripts, 35,182 (32.5%) carried at least one heterozygous site; of which 15,600 contained only one heterozygous site, 17,564 had 2 to 10 heterozygous sites and 2,018 had 11 to 59 heterozygous sites.

### Annotation of chrysanthemum unique transcript sequences

We first annotated the assembled chrysanthemum unique transcripts through homologous search against different protein databases. A total of 58,083 (59.2%), 41,691 (42.5%), 57,975 (59.1%) and 55,463 (56.5%) unique transcripts had significant hits (E-value ≤ 1e-5) in the GenBank non-redundant (nr), Swiss-Prot, TrEMBL and Arabidopsis protein databases, respectively. Consistent with previous reports, we also observed that the percentage of genes that can be annotated was positively correlated with the length of genes (Figure 1).

**Figure 2:**
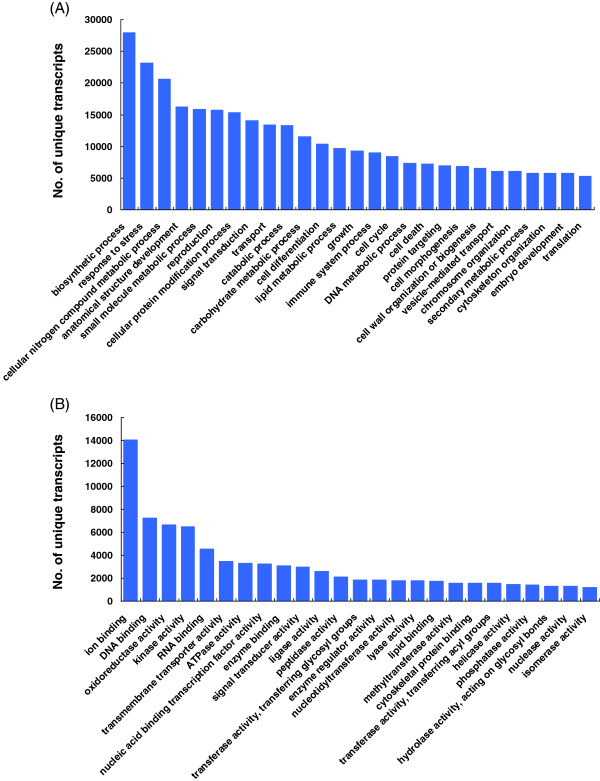
**Functional classification of chrysanthemum unique transcripts. (A)**, Within the category of biological process. **(B)**, Within the category of molecular function.

We further annotated the chrysanthemum unique transcripts by assigning them with gene ontology (GO) terms. A total of 52,297 unique transcripts (53.2%) were assigned with at least one GO term, among which 48,175 (49.1%) were assigned in the biological process category, 45,804 (46.7%) in the molecular function category, and 45,168 (46.0%) in the cellular component category, while 39,200 (39.9%) unique transcripts were assigned GO terms in all three categories. We then further classified the chrysanthemum unique transcripts into different functional categories using a set of plant-specific GO slims (http://www.geneontology.org/GO.slims.shtml). The top 25 groups in the biological process and molecular function categories are shown in Figure 2. It is worth mentioning that “response to stress” represented the second most abundant group in the biological process category, only after “biosynthetic process”, consistent with the fact that our transcriptome data were derived from chrysanthemum plants under dehydration stress. Other interesting highly abundant groups in the biological process category included small molecule metabolic process, signal transduction, transport, catabolic process and cell differentiation (Figure 2A). In the category of molecular function, the most abundant groups included ion binding, DNA binding and oxidoreductase activity, and other appealing groups included kinase activity, transmembrane transporter activity, nucleic acid binding transcription factor activity and signal transducer activity (Figure 2B).

**Figure 3:**
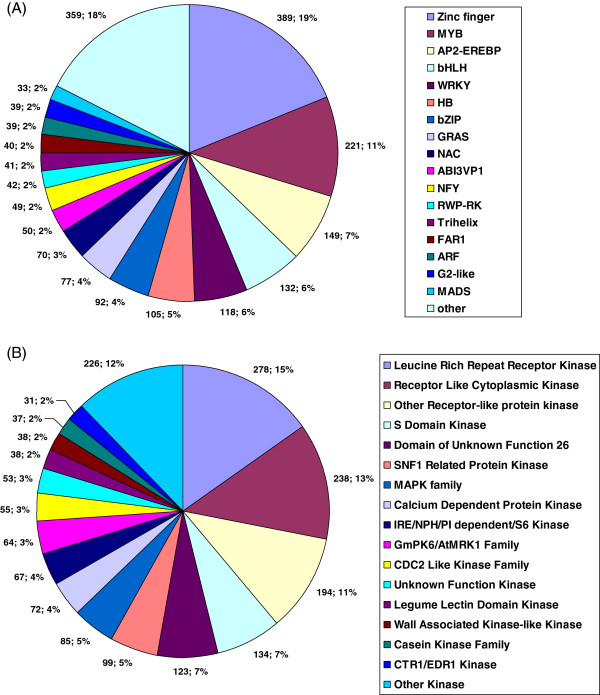
**Number of unique transcripts annotated as transcription factor and protein kinase in chrysanthemum transcriptome sequences. (A)**, Transcription factor. **(B)**, Protein kinase.

Transcription factors and protein kinases are important upstream regulatory proteins and play critical roles in various plant developmental processes and plant responses to abiotic and biotic stresses. In the present study, from the chrysanthemum unique transcripts, we identified a total of 2,028 transcription factors that were classified into 55 different families and 1,832 protein kinases set into 65 different families (Figure 3). The largest group of transcription factors was the MYB family (221, 10.9%), followed by C3H (169, 8.3%), AP2-EREBP (149, 7.3%), C2H2 (136, 6.7%), bHLH (132, 6.5%), and the WRKY (118, 5.8%) families (Figure 3A). These six families represented approximately half of the transcription factors identified in the unique chrysanthemum transcripts. The most abundant group of protein kinases was receptor-like protein kinase family (710, 38.8%), which included leucine-rich repeat receptor kinases (278, 15.2%), receptor like cytoplasmic kinases (238, 13.0%) and other receptor-like protein kinases (194, 10.6%). Other abundant groups included S-domain kinase (134, 7.3%), domain of unknown function 26 (DUF26) kinase (123, 6.7%) and SNF1-related protein kinase (99, 5.4%) families (Figure 3B). We also found that protein kinases such as MAPKs (85, 4.6%) and calcium dependent protein kinases (72, 3.9%), which have been reported to play important roles in plant responses to dehydration stress [19,20], were also highly abundant in our transcriptome dataset.

We further predicted biochemical pathways from the assembled chrysanthemum transcripts. A total of 366 pathways represented by 10,566 unique enzyme-encoding transcripts were obtained. It is known that chrysanthemum contains various valuable secondary metabolites, biologically active compounds and essential oils, and some of them such as flavonoid and chlorogenic acid have important medicinal functions [1]. In this study, most of the secondary metabolites pathways such as flavonoid biosynthesis, chlorogenic acid biosynthesis, methylquercetin biosynthesis, ergosterol biosynthesis, avenacin biosynthesis and linear furanocoumarin biosynthesis were well covered by our unique transcripts. In addition, abiotic stress-related pathways such as proline and trehalose biosynthesis pathways were also identified and most enzymes in these pathways were found in our chrysanthemum transcript dataset.

### Dynamic transcriptome profiles under dehydration in chrysanthemum

**Figure 4:**
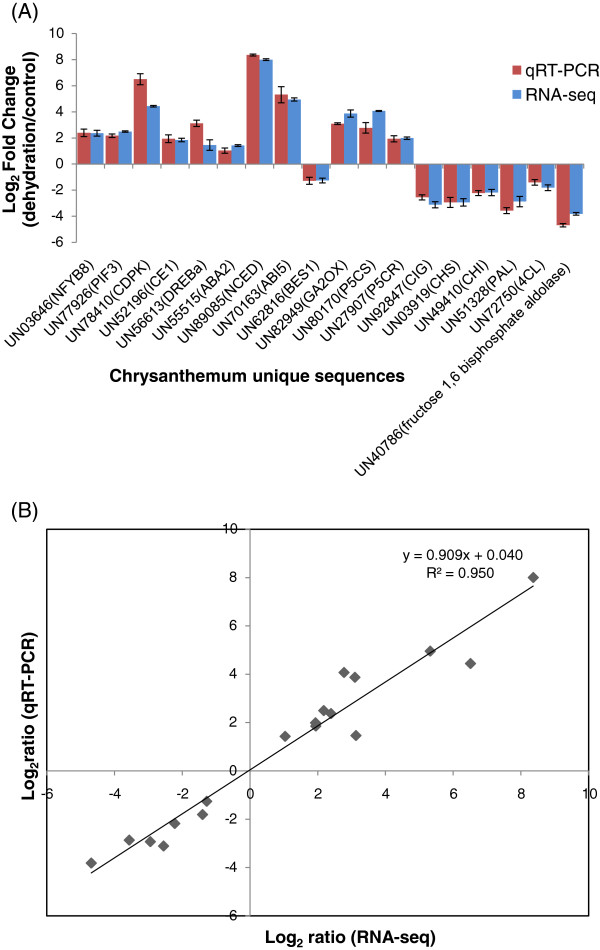
**Verification of RNA-seq results by qRT-PCR.** Eighteen unique transcripts with significantly altered expression pattern in response to dehydration were selected from transcription factors, signal components, and biochemical pathways. **(A)**, Comparison of expression level of unique transcripts between RNA-seq and qRT-PCR. Primers for qRT-PCR are listed in Additional file 4. **(B)**, Scatter diagram of log ratios (Log_2_ FC) of unique transcripts. qRT-PCR data were normalized using the ‘housekeeping’ gene *CmUBI*.

We first calculated correlation coefficients of transcriptome profiles among the six samples and between the technical replicates (Table 2). The high correlation among biological replicates and between technique replicates indicated the robustness of our RNA-seq dataset. In this study, we only used reads of 100 bp for expression profile analysis. To further validate our RNA-seq expression profile data, we performed qRT-PCR assays on eighteen selected drought-responsive unique transcripts. The results showed that although the exact fold changes of the selected unique transcripts varied between RNA-seq expression and qRT-PCT analyses (Figure 4A), the high correlation (R^2^ = 0.95) described by a simple linear regression equation y = 0.96× + 0.40 (Figure 4B) indicated the good consistency between the two analysis techniques.

**Table 2:** Correlation coefficients of transcriptome profiles among RNA-seq samples

	**T1**	**T2**	**T3**	**T1-1**	**T2-1**	**T3-1**	**CK1**	**CK2**	**CK3**	**CK1-1**	**CK2-1**	**CK3-1**
T1	1	0.98	0.97	0.98	0.96	0.96	0.54	0.58	0.57	0.55	0.58	0.58
T2	0.98	1	0.99	0.98	0.99	0.98	0.57	0.59	0.59	0.58	0.6	0.6
T3	0.97	0.99	1	0.97	0.98	0.99	0.58	0.61	0.61	0.59	0.62	0.62
T1-1	0.98	0.98	0.97	1	0.99	0.98	0.5	0.53	0.52	0.51	0.54	0.54
T2-1	0.96	0.99	0.98	0.99	1	0.99	0.53	0.55	0.55	0.54	0.56	0.56
T3-1	0.96	0.98	0.99	0.98	0.99	1	0.55	0.57	0.57	0.56	0.58	0.59
CK1	0.54	0.57	0.58	0.5	0.53	0.55	1	1	1	1	0.99	0.99
CK2	0.58	0.59	0.61	0.53	0.55	0.57	1	1	1	0.99	1	1
CK3	0.57	0.59	0.61	0.52	0.55	0.57	1	1	1	0.99	0.99	1
CK1-1	0.55	0.58	0.59	0.51	0.54	0.56	1	0.99	0.99	1	1	0.99
CK2-1	0.58	0.6	0.62	0.54	0.56	0.58	0.99	1	0.99	1	1	1
CK3-1	0.58	0.6	0.62	0.54	0.56	0.59	0.99	1	1	0.99	1	1

T1, T2, T3: dehydration treated samples sequenced at 100 bp.

T1-1, T2-1, T3-1: dehydration treated samples sequenced at 50 bp.

CK1, CK2, CK3: control samples sequenced at 100 bp.

CK1-1, CK2-1, CK3-1: control samples sequenced at 50 bp.

In total, we identified 8,558 differentially expressed unique transcripts between dehydration-treated and control plants, among which 5,371 were induced by dehydration stress and 3,187 repressed. We then identified GO terms in the biological process category that were significantly enriched in dehydration-induced and repressed genes, respectively (Additional file 1). As expected, GO terms including “response to water deprivation”, “response to water stimulus”, “response to desiccation”, “response to osmotic stress” were highly enriched in both dehydration-induced and repressed genes. This further validated the efficiency of our dehydration stress treatments and the reliability of our gene expression data. In addition, GO terms related to responses to various other types of abiotic stresses such as salt, high temperature and cold were also highly enriched in dehydration-responsive genes, indicating the crosstalk of different stress responses in chrysanthemum, same as those reported in other plant species [21].

Plant hormones are known to be involved in plant responses to various stresses. In this study, we found that GO terms including “response to abscisic acid stimulus”, “response to salicylic acid stimulus”, “response to jasmonic acid stimulus” and “response to ethylene stimulus” were highly enriched in both dehydration up- and down-regulated genes, whereas “response to brassinosteroid stimulus”, “response to auxin stimulus” and “response to cytokinin stimulus” were only enriched in genes repressed by dehydration. Interestingly, we found the biosynthetic processes of jasmonic acid and salicylic acid were down-regulated, which are usually up-regulated by dehydration stress in other plant species [22,23], suggesting that chrysanthemum may have different hormone regulatory mechanisms in response to dehydration. These results indicated that almost all plant hormones were involved in chrysanthemum response to the dehydration stress, and the hormones might play different roles in chrysanthemum compared to other plant species, and the crosstalk among different hormones existed in chrysanthemum in response to dehydration.

Plants can accumulate secondary metabolites such as phenylpropanoids, terpenoids and flavonoid under the dehydration stress condition, and these metabolites function as potent scavengers of reactive oxygen species [24]. However, in this study we found GO terms including “secondary metabolite biosynthetic process”, “flavonoid biosynthetic process”, “flavonoid metabolic process”, “xanthophyll biosynthetic process” and “anthocyanin-containing compound biosynthetic process” were highly enriched in dehydration down-regulated genes. The down-regulation of these second metabolisms might be largely due to the decrease of the jasmonate biosynthesis, because JA acts as a conserved elicitor of plant secondary metabolism [25].

Interestingly, the GO term “response to karrikin” was found to be significantly enriched in dehydration-repressed genes. Karrikins are a new group of plant growth regulators found in the smoke of burning plant materials, that can trigger Arabidopsis seed germination [26]. Recently it is revealed to be involved in the response to cold stress in *Celtis bungeana* and Arabidopsis [27]. Currently the biological and molecular functions of karrikins are still unknown. Our results suggested that karrikins might play important roles in dehydration tolerance in chrysanthemum.

To our surprise, GO terms “response to red or far red light” and “regulation of short-day photoperiodism, flowering” were highly enriched in dehydration down-regulated genes, while “regulation of long-day photoperiodism, flowering” was found to be enriched in dehydration up-regulated genes. These results suggest that chrysanthemum as a short-day plant species can elevate vegetative growth through delaying flowering time to avoid dehydration-caused damages [28,29]. In addition, since photoperiod can regulate the DREB/CBF pathway [30], our data suggested that photoperiod related genes might also regulate dehydration responses in chrysanthemum.

### Dehydration-responsive transcription factors and protein kinases in chrysanthemum

**Figure 5:**
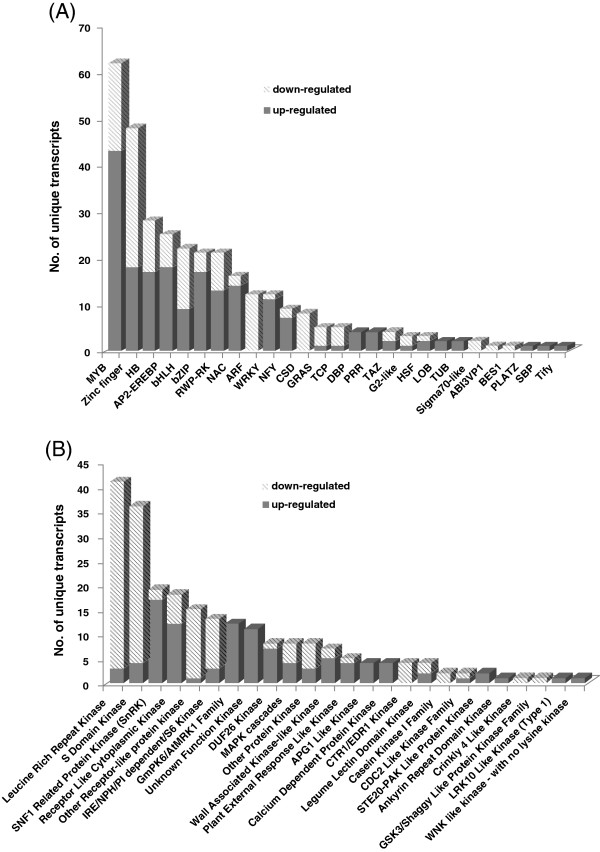
**Number of unique transcripts annotated as transcription factor and protein kinase in response to dehydration stress. (A)**, Transcription factor. **(B)**, Protein kinase.

Transcription factors and protein kinases are important upstream regulators of plant responses to various biotic and abiotic stresses. In the present study, we identified a total of 306 transcription factors and 228 protein kinases that were responsive to dehydration stress in chrysanthemum. These transcription factors and protein kinases were classified into 27 and 25 families based on their putative DNA binding and kinase domains, respectively (Figure 5 and Additional file 2).

Among the dehydration-responsive transcription factor families, the MYB family was the largest group, containing 62 unique transcripts (43 up-regulated and 19 down-regulated), followed by the Zinc finger (18 and 30), the AP2/EREBP (18 and 7) and the HB (17 and 9) families. Genes from these families have been reported to play significant roles in plant responses to various environmental stresses in other plant species [31].

It is well known that plants possess both ABA-independent and ABA-dependent regulatory systems to control drought-inducible gene expression [3]. DREB (also known as CBF) genes play an important role in regulating ABA-independent gene expression in response to drought [32]. In the present study, a total of eight DREB genes belonging to the AP2/EREBP family were found to be responsive to dehydration in chrysanthemum. In addition, an ICE1 (inducer of CBF expression 1) homolog (UN52196) in the bHLH family was found to be induced by dehydration in chrysanthemum. However, ICE1 in Arabidopsis is induced by cold stress but not by dehydration [33], indicating possible different mechanisms of dehydration responses between chrysanthemum and Arabidopsis.

In this study, we found six basic leucine zipper (bZIP) family genes encoding AREB (ABA-responsive element binding) and ABI5-like (abscisic acid-insensitive 5-like) proteins that were up-regulated by dehydration stress in chrysanthemum. These genes are known to be induced by ABA and involved in drought stress response in an ABA-dependent manner in other plant species [34]. TOC1 (timing of CAB expression 1) is known to function in the core loop of the clock and controls a suite of clock genes and clock output genes [35]. Recent studies revealed that the expression of *TOC1* is induced by ABA and TOC1 functions as a molecular switch connecting the circadian clock with ABA signaling in response to drought in Arabidopsis [36]. In this study, we found four PRR family genes including a TOC1 homologue (UN37013) that were up-regulated by dehydration, suggesting that this family genes might also play important roles in chrysanthemum response to dehydration.

Plant NF-Y (Nuclear Factor Y), which binds to the *cis*-element CCAAT, is an important regulator that can coordinate plant responses to drought stress [37,38]. NF-Y is a conserved heterotrimeric complex consisting of NF-YA, NF-YB, and NF-YC subunits [39]. In this study, we identified three transcripts encoding NF-YA subunits and four encoding NF-YB subunits that were up-regulated by dehydration and two encoding NF-YC subunits that were down-regulated.

Among the differentially expressed protein kinases, Leucine Rich Repeat (LRR) kinase (3 up-regulated and 38 down-regulated) was the largest family, followed by S domain kinase (4 and 32) and SNF1-related protein kinase (17 and 2) families. LRR kinases, which belong to the protein receptor kinase (PRK) family, are key components in the mediation of plant responses to dehydration [40]. S domain kinase family proteins are single-pass transmembrane Ser/Thr kinases, which are known to determine plant self-incompatibility [41]. S domain kinases are also up-regulated by pathogen infection and wounding or salicylic acid (SA) [42]. However, up to now, there is little information regarding the response of S domain kinases to dehydration stresses. SNF1-related protein kinases (SnRKs) might sense the ATP/AMP ratio and thus regulate fluxes between anabolism and catabolism, and also play an important role in the ABA signaling in response to drought stress [8].

We also found that the expression of eight MAPKs and four calcium dependent protein kinases (CDPKs) were affected by dehydration stress in chrysanthemum. It has been reported that MAPKs and CDPKs can be activated in response to drought and other abiotic stresses as a link between stress sensor and pathway targets [8].

### Dehydration-induced biochemical changes in chrysanthemum

In order to assess the functional roles of dehydration-responsive genes involved in different biochemical pathways, and to study the biochemical adaptations to dehydration stress in chrysanthemum, we identified biochemical pathways affected by dehydration stress based on our expression profiling analysis. A total of 58 biochemical pathways that were significantly affected by dehydration stress (p value < 0.05) were identified (Additional file 3). These pathways covered the biosynthesis or degradation of diverse metabolites including hormones, sugars and polysaccharides, amino acids, fatty acids and lipids, and secondary metabolites, indicating a comprehensive impact of dehydration stress on chrysanthemum growth and metabolism.

Dehydration stress is previously known for producing high levels of toxic reactive oxygen intermediates (ROIs), but recent studies also determine the function of ROIs as an integral cellular signaling molecules [43]. Plants have developed an antioxidant system to remove the excess superoxide radicals. This antioxidant system contains several important enzymes including superoxide dismutase (SOD), ascorbate peroxidase (APX) and catalase (CAT) [44]. In the present study, five transcripts encoding CATs (UN17383, UN23112, UN68661, UN97450 and UN97695) were up-regulated by dehydration stress while one encoding chloroplast Fe SOD (UN52726) was down-regulated. Four unique transcripts encoding glutathione S-transferase were down-regulated by dehydration. These results suggest that dehydration may trigger the complex antioxidant network, and finely tuned ROS accumulation to facilitate appropriate signaling functions [45].

**Figure 6:**
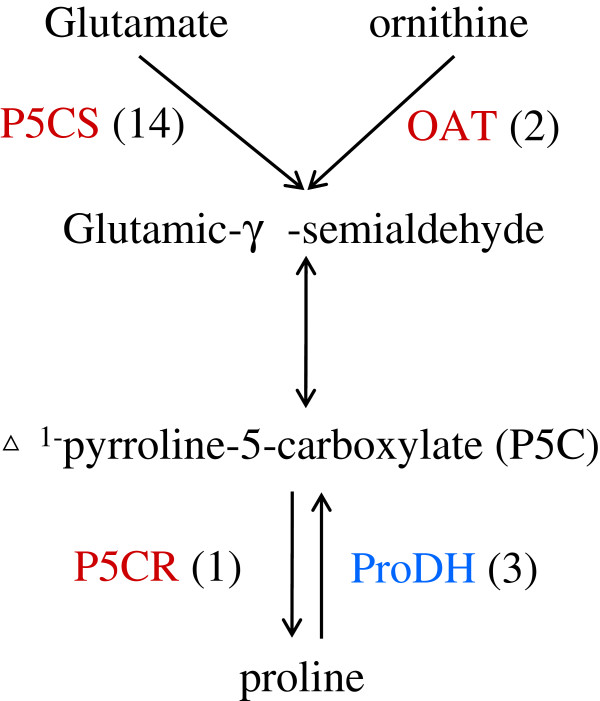
**Proline biosynthetic pathway in chrysanthemum under dehydration.** P5CS, pyrroline-5-carboxylate synthetase; P5CR: pyrroline-5-carboxylate reductase; ProDH, proline dehydrogenase; OAT, ornithine aminotransferase. Genes in red and blue mean up- and down-regulated by dehydration, respectively. Numbers in brackets represent numbers of unique transcript regulated by dehydration stress.

Plants accumulate prolines which function as osmolytes to stabilize cell proteins and structures under stresses. Proline is also considered as a scavenger of free radicals, an energy sink and a stress-related signal [46]. Proline plays a role in cellular homeostasis, specifically at redox balance and energy status in Arabidopsis [47]. Recently, a new role of tissue-specific proline synthesis and proline catabolism has been found in promoting growth and maintaining a higher NADP/NADPH ratio at low water potential [48]. In the present study, all unique transcripts in the proline biosynthetic pathway were found to be up-regulated by dehydration, especially those encoding the key enzyme pyrroline-5-carboxylate synthetases (P5CS) which showed 23–26 fold increases of their expression. On the contrary, transcripts encoding proline dehydrogenase (ProDH), the key enzyme in the proline degradation pathway, were down-regulated (Figure 6). We therefore further determined the expression changes of three key enzyme genes (*P5CS*, *P5CR* and *ProDH*) in proline biosynthetic and degradative pathways under dehydration (Table 3). Surprisingly, expression of *P5CS* and *P5CR* was down-regulated at 1 h time point of dehydration treatment and then swiftly up-regulated after 3 h dehydration treatment, while *ProDH* depicted an opposite expression level to that of *P5CS* and *P5CR*. The results suggested that proline catabolism in chrysanthemum may be needed to support plant growth during mild dehydration, and proline biosynthesis may help to maintain cellular redox balance just like Tsu-1/Kas-1 example [49]. In addition, we found that unique transcripts in the stachyose and trehalose biosynthesis pathways were also up-regulated by dehydration in chrysanthemum. The accumulation of these osmolytes could be critical to improve osmotic stress tolerance of chrysanthemum.

**Table 3:** Expression changes of unique transcripts related to biochemical pathways in response to dehydration in chrysanthemum

**Accession no.**	**Relative expression level**				
	**Dehydration time (fresh weight loss)**				
	**0 h (100%)**	**1 h (18%)**	**3 h (39%)**	**6 h (53%)**	**12 h (72%)**
UN89085(NCED)	1.00 ± 0.07	0.83 ± 0.09	329.49 ± 16.43	59.89 ± 3.68	16.84 ± 0.52
UN55515(ABA2)	1.00 ± 0.00	1.31 ± 0.14	2.16 ± 0.29	1.89 ± 0.46	2.20 ± 0.32
UN80170(P5CS)	1.00 ± 0.16	0.42 ± 0.05	7.46 ± 1.91	6.23 ± 1.00	2.05 ± 0.51
UN27907(P5CR)	1.00 ± 0.18	0.72 ± 0.08	4.14 ± 0.63	3.24 ± 0.15	4.00 ± 0.26
UN92847(ProDH)	1.00 ± 0.29	1.72 ± 0.04	0.17 ± 0.02	0.31 ± 0.03	0.11 ± 0.02
UN51328(PAL)	1.00 ± 0.04	1.21 ± 0.15	0.09 ± 0.01	0.26 ± 0.05	0.10 ± 0.00
UN72750(4CL)	1.00 ± 0.08	0.85 ± 0.08	0.36 ± 0.06	0.42 ± 0.10	0.29 ± 0.03
UN03919(CHS)	1.00 ± 0.07	0.97 ± 0.03	0.12 ± 0.04	0.03 ± 0.00	0.03 ± 0.01
UN49410(CHI)	1.00 ± 0.05	1.08 ± 0.15	0.21 ± 0.03	0.13 ± 0.06	0.08 ± 0.02

Unique transcripts in the table were selected from changed biochemical pathways in Additional file 3.

Lipids are known to function as the structural constituents of most cellular membranes and can be oxidized through the 13-lipoxygenase (13-LOX) and 13-hydroperoxide lyase (13-HPL) pathway [50]. Same as in Arabidopsis [51], the 13-LOX and 13-HPL pathway was significantly down-regulated under the dehydration condition in chrysanthemum, which could maintain the membrane integrity to reduce damages caused by drought.

**Figure 7:**
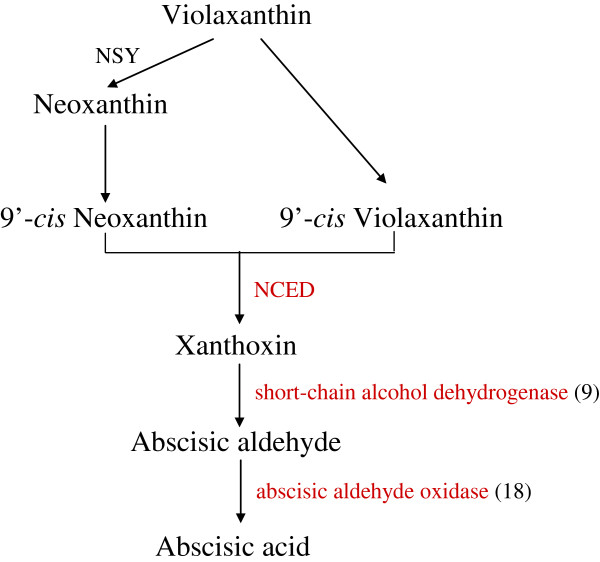
**ABA biosynthetic pathway in chrysanthemum under dehydration.** NSY: neoxanthin synthase; NCED: 9-cis-epoxycarotenoid dioxygenase. Genes in red mean up-regulated by dehydration. Numbers in brackets represent numbers of unique transcript regulated by dehydration stress.

ABA is essential for various stress responses and the endogenous ABA level plays a key role in such ABA-dependent stress responses [46]. In this study, we found that key enzymes of ABA biosynthesis such as 9-cis-epoxycarotenoid dioxygenase (NCED), short chain alcohol dehydrogenase and abscisic-aldehyde oxidase were all up-regulated by dehydration in chrysanthemum (Figure 7). The expression of some NCED transcripts (UN53982, UN89085 and UN31557) was induced by nearly 200 folds. These changes in gene expression were further confirmed through qRT-PCR analysis in response to dehydration (Table 3). These results suggested that the dehydration stress highly elevated the biosynthesis of ABA and activated the ABA-dependent pathway in chrysanthemum.

Bioactive gibberellins (GAs) control diverse aspects of growth and development, including seed germination, stem elongation, leaf expansion, and flower and seed development [52]. We found that one transcript (UN97230) encoding ent-kaurenoic acid oxidase, the key enzyme of GA biosynthesis, was down-regulated by dehydration, while a transcript encoding GA 2-oxidase (GA2ox) in the GA deactivation pathway was up-regulated. These might lead to the decreased endogenous level of bioactive GA just like other plant species [53]. In addition, we found that the biosynthetic pathways of SA, JA and BR were also affected by the dehydration stress. Cross-talk between the different plant hormones results in synergetic or antagonistic interactions that play crucial roles in response of plants to abiotic stress [54]. The changes of the levels of these hormones might together coordinate the regulatory network of the stress signaling and dehydration tolerance in chrysanthemum.

Our data also showed that both photosynthesis and glycolysis of chrysanthemum plants were restrained by dehydration, which led to the decreased level of plant energy usage. Fructose 1, 6-bisphosphate aldolase, the key enzyme in the gluconeogenesis pathway, were down-regulated under dehydration stress in chrysanthemum, which was a similar phenomena found in Arabidopsis and tomato [55]. Gluconeogenesis consumes a lot of energy, thus decreased activities of gluconeogenesis can be considered as a self-protection mechanism for plants to save energy under dehydration conditions.

We also found that key enzymes related to some important secondary metabolisms were significantly down-regulated by dehydration, including chalcone synthase (CHS) and chalcone isomerase (CHI), the key enzymes in the flavonoid biosynthesis pathway, zeaxanthin epoxidase and violaxanthin deepoxidase, the key enzymes in the antheraxanthin and violaxanthin biosynthesis pathway and xanthophyll cycle pathway, hydroxycinnamoyl CoA quinate transferase, and one of the key enzymes in the chlorogenic acid biosynthesis pathway. We further investigated the expression profiles of four unique transcripts encoding key enzymes involved in flavonoid biosynthesis (*phenylalanine ammonia-lyase*, *4-coumarate: coenzyme A ligase*, *CHS*, and *CHI*) during dehydration. Expression of these unique transcripts was clearly down-regulated after 3 h dehydration treatment as compare to control plants (Table 3). As defensive compounds (secondary metabolism) are exhaustive in nature and their accumulation may take place at the expense of plant’s growth [56]. Down-regulation of the secondary metabolite biosynthesis by dehydration in chrysanthemum could be considered as a protection strategy against dehydration-caused damages through saving energy and carbon supply to maintain plant survival under the quick water loss condition.

In summary, our data suggested that the regulation of various biochemical pathways may help plants to cope with drought stresses, mainly through regulating hormone signaling, reducing oxidative damage, stabilizing cell proteins and structures, and maintaining energy and carbon supply.

## Conclusions

In the present study, we performed large-scale transcriptome sequencing of chrysanthemum plants under dehydration stress using the Illumina sequencing technology. A total of more than 100 million reads were generated and de novo assembled into 98,180 unique transcripts which were further extensively annotated by comparing their sequences to different protein databases.

We also performed gene expression profiling analysis upon dehydration treatment in chrysanthemum and identified 8,558 dehydration-responsive unique transcripts, including 307 transcription factors and 229 protein kinases and many well-known stress responsive genes. Gene ontology (GO) term enrichment and biochemical pathway analyses showed that dehydration stress caused changes in hormone response, secondary and amino acid metabolism, and light and photoperiod response. These findings suggest that drought tolerance of chrysanthemum plants may be related to the regulation of hormone biosynthesis and signaling, reduction of oxidative damage, stabilization of cell proteins and structures, and maintenance of energy and carbon supply.

Collectively, our transcriptome sequences can provide a valuable resource for chrysanthemum breeding and research and novel insights into chrysanthemum responses to dehydration stress and offer candidate genes or markers that can be used to guide future efforts attempting to breed drought tolerant chrysanthemum cultivars.

## Methods

### Plant material and stress treatment

Chrysanthemum (*Chrysanthemum morifolium*) ‘Fall Color’, a popular ground-cover type cultivar with pink color flowers was used in this study. Plant cultivation was performed as described previously [57]. Prior to the treatment, roots were washed, carefully prevented from mechanical damage, and then placed in distilled water for 12–24 h. Both dehydration and control plants were placed under same growing conditions with 22°C temperature, 40%–50% relative humidity and continuous light (100 μmol m^-2^ s^-1^).

**Figure 8:**
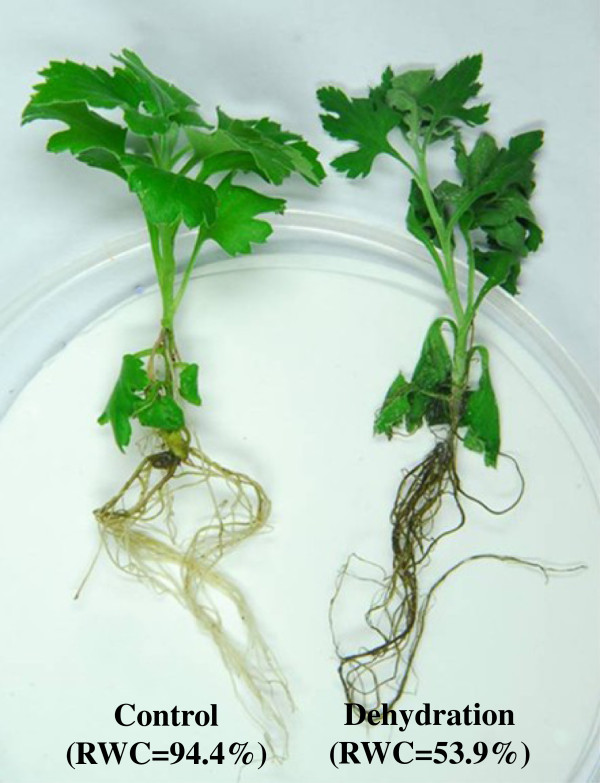
Chrysanthemum plants under dehydration and well-watered conditions.

For sampling of RNA-seq, the plants were exposed to air-drying on a filter paper for 3 h, and the control plants were still kept in distilled water (Figure 8). Relative water content (RWC) of samples was accordingly measured [58].

For gene expression profiles, the plants were exposed to air-drying on a filter paper for time intervals of 1 h, 3 h, 6 h and 12 h, respectively. Fresh weight was measured during dehydration treatment.

Leaves and roots samples were then collected from the control and dehydration-treated plants and immediately frozen in liquid nitrogen and stored at −80°C till use.

### Total RNA extraction, RNA-seq library construction and sequencing

Total RNA was extracted using the RNeasy Plant Mini kit (Qiagen, Valencia, CA) following the manufacturer’s instruction. Strand-specific RNA-seq libraries were constructed as previously described [59] and sequenced on a HiSeq2000 system according to the manufacturer’s instructions in the core facility of Cornell Weill Medical College. Three biological replicates were sequenced for each treatment and two technique replicates were also performed for each sample, with one sequenced at 51 bp and the other at 100 bp. The raw sequence reads were deposited into NCBI SRA database under accession no. SRA091277.

### RNA-seq data processing, de novo assembly and annotation

RNA-seq reads were first processed with a custom R script based on the ShortRead package [60] to trim low quality (Q value < 20) nucleotides on both ends and to clip the adapter and barcode sequences from the 3’ end. The resulting reads with length less than 40 bp or containing more than two ambiguous (“N”) nucleotides were discarded. The RNA-seq reads were then aligned to GenBank virus (version 186) and the ribosomal RNA (rRNA) sequence databases using BWA [61] using default parameters. Reads mapped to these two databases were discarded. The resulting high-quality cleaned reads were assembled *de novo* into contigs using Trinity with strand specific option “--SS_lib_type” set to “F” and “min_kmer_cov” set to 2 [62]. To remove the redundancy of Trinity-generated contigs, they were further assembled de novo using iAssembler with minimum percent identify (−p) set to 99 [63].

The resulting unique transcripts were blasted against GenBank non-redundant (nr), UniProt (Swiss-Prot and TrEMBL), and Arabidopsis protein databases with a cutoff E-value of 1e-5. Gene ontology (GO) terms were assigned to the chrysanthemum assembled transcripts based on the GO terms annotated to their corresponding homologues in the UniProt database. Biochemical pathways were predicted from the chrysanthemum transcripts using the Pathway Tools [64]. Transcription factors (TF) and protein kinases were identified and classified into different families using the iTAK pipeline (http://bioinfo.bti.cornell.edu/tool/itak).

### Identification of chrysanthemum heterozygous sites

To identify heterozygous sites in the chrysanthemum transcripts, the cleaned reads were first aligned to the assembled transcript sequences using BWA allowing one mismatch and with the seed region set to 50 bp. Following alignments, the coverage of each position on the transcripts by base A, G, C and T was calculated. Loci containing at least two genotypes with each of them supported by at least five reads and allele frequency of at least 0.1 were identified as heterozygous sites.

### Gene expression quantification and differential expression analysis

We aligned the high-quality cleaned RNA-seq reads to the assembled chrysanthemum transcripts with the Bowtie program [65] allowing one mismatch. Following alignments, raw counts for each chrysanthemum transcript and in each sample were derived and normalized to reads per kilobase of exon model per million mapped reads (RPKM). For differential expression analysis, only samples with RNA-seq reads of 100 bp in length were used. Differentially expressed genes (fold changes > 2 and adjusted p-value < 0.001) between normal and dehydration-treated conditions were identified with the DESeq package [66]. GO terms enriched in the set of differentially expressed genes and pathways that were affected by drought treatment were identified using the Plant MetGenMAP system [67].

### Quantitative RT-PCR analysis

For quantitative RT-PCR of mRNAs, 1 μg DNase I-treated total RNA was used to synthesize cDNA by M-MLV (Promega) using poly(dT)18 oligonucleotides. *CmUBI* was used as an internal control. qRT-PCR was performed using KAPA™ SYBR® FAST qPCR kits (Kapa Biosystems, Woburn, MA) on a StepOne Plus Real Time PCR System (Applied Biosystems, Foster City, USA) according to the manufacturer’s instruction. Products were verified by melting curve analysis. Quantification was achieved by normalizing the number of target transcripts copies to the reference *CmUBI* gene using the comparative ΔΔCt method [68]. All reactions were performed with at least three biological replicates. Primers used in all quantitative RT-PCR experiments are listed in Additional file 4.

### Availability of supporting data

The datasets supporting the results of this article are available in Chrysanthemum Transcriptome Database repository [http://www.icugi.org/chrysanthemum], and in the NCBI SRA repository [http://www.ncbi.nlm.nih.gov/sra?term = SRA091277].

## Competing interests

The authors declare that they have no competing interests.

## Authors’ contributions

YX and SG performed the sequence analysis. YY prepared RNA samples for RNA sequencing. MH provides aids in the data analysis, management and website uploading. LC and QW helped with data interpretation. BH, ZF and JG designed the experiment and provided guidance on the whole study. All authors have read and approved the manuscript.

## Supplementary Material

Additional file 1 List of GO terms significantly enriched in genes that were up- or down-regulated by dehydration in chrysanthemum. Click here for file

Additional file 2 List of chrysanthemum dehydration-responsive transcription factors and protein kinases. Click here for file

Additional file 3 List of biochemical pathways that were significantly affected by dehydration stress in chrysanthemum. Click here for file

Additional file 4 Primer information of unique transcripts for the qRT-PCR analysis. Click here for file
