# Supplementary material for: Transcriptome sequencing and whole genome expression profiling of chrysanthemum under dehydration stress
Source: BMC Genomics. 2013 Sep 28;14:662. doi: 10.1186/1471-2164-14-662 (PMC3849779; doi:10.1186/1471-2164-14-662)
Supplement: Additional file 4 — Primer information of unique transcripts for the qRT-PCR analysis. [file 1471-2164-14-662-S4.docx]

Additional file 4: Primer information of unique transcripts for the qRT-PCR analysis

| **Accession No.** | **Annotation** | **Primer set** | |
| --- | --- | --- | --- |
|  |  | **Forward primer (5’-3’)** | **Reverse primer (5’-3’)** |
| UN03646 | NFYB | AGCTCGAGTCTTGTCTTTCAACT | AACTCACAGCTTGACAGCGT |
| UN80170 | P5CS | CGCAACCCCACTCTAACCAT | AGCAGTTCCTACCTTAACGACA |
| UN62816 | BES1/BZR1 | TTTGTGCTGAAGCTGGTTGG | TGGTTTGGCGGGTTTTCACA |
| UN70163 | ABI5 | GCAAAGTAGCAGCCGAAACC | TCAGCTTGGGAGTCCTGCTA |
| UN78410 | CDPK | ACGAAGCTACCAACAACTAACCT | TTACCAGGGGTCACACAACAA |
| UN56613 | DREBa | ATGCCTCACTTCACAAACCCA | GCCGCATAAATATCAAGCCCT |
| UN52196 | ICE1 | CCCCAGGAAGCCAAATGTCTAA | CGAGCTGAGCAATGCAAAGAA |
| UN77926 | PIF3 | TGGGATGATTCACAAACGGAG | ACCAGTCTGCTTTGGTTGACA |
| UN82949 | GA2ox | AGAAGGTGTTTGGATTCGGGTT | TGGTAACTATGCCCAAACCTCC |
| UN40786 | fructose 1,6 bisphosphate aldolase | CAACAAACCAAGCACTCTCCA | GGGCCAGTGCTAACTCACTT |
| UN51328 | PAL | GCACATTGCAAACAAATTCAAACT | CGAGTGTGTTGAGGGTTGGA |
| UN49410 | CHI | TCCATACCTCTCACACCTGC | AACTGTACTTACTACCATGGCAAC |
| UN03919 | CHS | ACACAATTAGCCGGAGTTGC | CACCGATATTAAACACCGATGGC |
| UN55515 | ABA2 | AGCAGCATGTTTCATCCCCA | ACAATGCTGGGATTGGAGGG |
| UN89085 | NCED | ACATGGATGGTGCGTGCAAT | AAGCTCAGCTGGTTTCCCTAA |
| UN27907 | P5CR | GCTTTCCATGTCTTGCCCAC | TGTCGGTAGTTGCTGGTGTC |
| UN72750 | 4-coumarate: coenzyme A ligase | TGCAAACAAGCTTTATCGCGT | CCACAATGGCGAATCCGTTC |
| UN92847 | ProDH | AACAAGCAAAGAGGTCCCCC | ATTAGCGGGGCGAAAAGGAT |
|  | CmUBI | AGCTGAGCAGACTCCCGATG | AGGCGAATCATCAGTACCAAGT |
